# Browse from Three Tree Legumes Increases Forage Production for Cattle in a Silvopastoral System in the Southwest Amazon

**DOI:** 10.3390/ani11123585

**Published:** 2021-12-17

**Authors:** Lucy Dablin, Simon L. Lewis, William Milliken, Alexandre Monro, Mark A. Lee

**Affiliations:** 1 Royal Botanic Gardens Kew, Richmond, London TW9 3AE, UK; w.milliken@kew.org (W.M.); a.monro@kew.org (A.M.); mark.lee@rhul.ac.uk (M.A.L.); 2 Department of Geography, University College London, London WC1E 6BT, UK; S.L.Lewis@ucl.ac.uk; 3 School of Environment, Earth and Ecosystem Sciences, Open University, Milton Keynes MK7 6AA, UK; 4 School of Geography, University of Leeds, Leeds LS2 9JT, UK; 5 Department of Health Studies, Royal Holloway, University of London, Egham TW20 0EX, UK

**Keywords:** agroforestry, silvopasture, sustainable, agriculture, livestock, biomass, intensification, browse, ecosystem

## Abstract

**Simple Summary:**

Unsustainable cattle ranching in the Amazon leads to land degradation and incentivizes deforestation. Planting trees in pastures (‘silvopasture’ or ‘silvopastoral systems’) is a novel approach that has the potential to increase the sustainability of cattle production in the Amazon. Trees provide additional feed whilst also enhancing biodiversity, capturing carbon and improving soil quality. We measured the potential contribution of tree forage to pasture-fed cattle at a trial farm in Peru. Three leguminous tree species (*Erythrina berteroana*, *Inga edulis* and *Leucaena leucocephala*) were planted with grass, and their productivity was compared to plots containing only grass. We compared destructive and non-destructive methodologies that estimated intake of tree forage by browsing cattle. We found that fresh tree foliage of the three tree species was palatable to cattle and could be directly browsed. Cattle mostly foraged below 1.6 m and consumed 99% of available foliage from *E. berteroana*, 75% of available forage from *I. edulis* and 80% of available forage from *L. leucocephala*. Plots containing trees and grass produced more forage biomass (mean > 2.2 Mg ha^−1^) than grass only plots (mean = 1.5 Mg ha^−1^). This research highlights the potential for sustainable intensification of livestock production in the Amazon.

**Abstract:**

Assessing the palatability of forage from locally adapted trees could improve the sustainability of livestock production systems. However, grasses continue to dominate livestock feed across the Amazon. We established a silvopastoral cattle farming system in Peru, comparing three different forage tree species with grass monocultures using a randomised block design. Trees were arranged in alleys of 0.5 × 7.5 m, planted alongside grass, and were directly browsed by cattle. Browse removal was estimated by three methods: destructive sampling, canopy measurements and leaf counts. We found that all three tree species were palatable to cattle. Plots containing trees and grass produced more available forage (mean > 2.2 Mg ha^−1^) for cattle than the grass monocultures (mean = 1.5 Mg ha^−1^). Destructive sampling below 1.6 m demonstrated that cattle consumed 99% of the available *Erythrina berteroana* forage, 75% of the available *Inga edulis* forage and 80% of the available *Leucaena leucocephala* forage in 8 days. This research demonstrates methodologies to estimate the intake of locally adapted browse species by cattle and highlights the potential benefits of silvopastoral systems in the Amazon. Planting trees could also benefit animal health and provide ecosystem services such as soil regeneration, enhanced nutrient cycling and carbon capture.

## 1. Introduction

Globally, natural forests and grasslands are declining due to human activities such as deforestation and urbanisation, an increased frequency of drought, fire and flooding, and changes in atmospheric CO_2_ concentrations [1]. As temperatures rise, the forage quality of grass is predicted to decline, which will reduce the productivity of livestock farms, with an associated increase in methane emissions from cattle [2]. In temperate regions, the potential of fodder derived from tree-crop systems are being explored to increase the sustainability of intensive livestock production (e.g., [3]). However, more studies are needed to assess how direct browsing of trees and shrubs in extensive livestock systems can enhance sustainable production. The expansion of cattle ranching in the Amazon has been a major driver of deforestation and land degradation [4,5]. The inclusion of trees and shrubs in livestock pastures is an agroforestry arrangement known as a ‘silvopastoral system’ or ‘silvopasture’. There is increased impetus to evaluate novel tree species that can contribute to resilient livestock production systems that both adapt to and mitigate climatic change and prevent further losses in biodiversity and forest cover [6]. The benefits of trees in pastures can include enhanced soil nutrient cycling and soil formation, increased rate of recovery of compacted soils, reduction in water and nutrient runoff and soil erosion, additional habitat to support biodiversity, mitigation of climate change via carbon sequestration and increased ecological connectivity [7,8,9,10]. In the tropics, trees planted in pastures also provide shade which can reduce heat stress. Heat stress in livestock has been associated with decreased feed intake and negative effects on production, reproductive health, milk yields, fitness and longevity [11,12,13,14].

Forage derived from trees and shrubs is known as browse. Browse can provide an important contribution to livestock diets [15,16]. Some tree forages have also shown potential to reduce ruminant methane emissions [17,18]. There are tree species native to the Amazon that are palatable to cattle and show potential for domestication in silvopastoral systems [19,20]. In the Bragantina region of Northern Brazil, Hohnwald et al. [21] demonstrated that the integration of woody species had the potential to enhance forage production, ecological stability and sustainability when compared to grass monocultures. However, it remains to be empirically demonstrated whether silvopasture in the Amazon could increase total forage production when compared to monoculture grass pasture.

This experiment evaluated forage production in a newly established silvopastoral farming system used for extensive cattle production in the Peruvian Amazon, where potential fodder trees are arranged in rows or ‘alleys’ available to cattle for direct browsing and inter-sown with pasture grass. Potential forage species were identified and characterized through discussions with local cattle workers who were familiar with browsing preferences, direct observation of cattle browsing, and secondary information gathered from existing literature. Three tree species were chosen, *Erythrina berteroana* Urb., *Inga edulis* Mart. and *Leucaena leucocephala* (Lam.) de Wit.

The relationship between plant chemistry and animal production is complex. For example, the widely planted forage tree legume *L. leucocephala* contains the toxic secondary compound mimosine in its leaves and pods [22]. Ruminant animals can be inoculated with microbes that are shown to be able to break down mimosine to non-toxic compounds. This suggests the existence of other rumen organisms capable of degrading other phenolic compounds [23]. Due to the multiple interactions between plants, chemistry, and livestock, chemical analysis alone cannot ascertain forage value or whether a forage will be palatable to animals [24]. Feeding trials may be considered the most appropriate way to ascertain palatability and the effect of forage on animal nutrition [25].

The chemical composition of *L. leucocephala* forage has been widely documented and the limited information available documenting the chemical composition of *E. berteroana* and *I. edulis* suggested that they may be suitable as animal forage. Flores et al. [26] determined that dry matter (DM) of four-month-old regrowth of *E. berteroana* had a chemical composition of 29.2% crude protein (CP), 58.5% neutral detergent fibre (NDF), 38.8% acid detergent fibre (ADF). They reported a DM digestibility in vitro after 96 h of incubation as 54.3%. Dechnik-Vazquez et al. [16] reported a bromatological analysis of *I. edulis* as 3.43% CP, 57.6% non-structural carbohydrates and 57.6% ADF. Garcia et al. [27] reviewed 65 publications on *L. leucocephala* and found that they reported median chemical composition for *L. leucocephala* forage to be 3.52 nitrogen, 22.03 CP, 0.26 phosphorous, 0.33 magnesium, 1.45 potassium and 881.6 mg kg^−1^ of oxalate (g 100 g^−1^ DM). Digestible energy ranged from 11.6 to 12.9 MJ kg^−1^ DM and the values for total apparent digested CP ranged from 64.7 to 78%. *L. leucocephala* leaf meal was found to be 4.3 mimosine and 1.01 tannin (g 100 g^−1^ DM).

There is a paucity of studies that attempt to estimate total available forage to directly browsing animals. Therefore, this study describes a series of methodologies that seek to estimate the amount of tree browse available to cattle and the amount of browse removed by direct browsing. This study sought to (i) determine whether three tree species, *E. berteroana*, *I. edulis* and *L. leucocephala* are palatable to cattle and consumed when fresh, (ii) empirically evaluate whether tree and grass arrangements can surpass the amount of edible biomass than that found in monocultures of the grass *Brachiaria brizantha* cv. Marandú and (iii) understand how foliage height may influence the availability of browse to directly browsing cattle.

## 2. Materials and Methods

### 2.1. Site and Experimental Design

The experiment was carried out on a private experimental station, 7 km from the town of Puerto Maldonado in Madre de Dios, Peru (12°32′22.1″ S, 69°10′21.4″ W; 182 m altitude). The site was a cattle pasture from 1960–2003 and left fallow until 2016 when the vegetation was cleared. The site was situated on recent alluvium soils, that demonstrated a 0–5 cm organic soil horizon, low cation exchange capacity, low base saturation and acid pH. The dominant mineral in the soil was kaolinite.

Each treatment contained only one species of tree and the pasture grass *Brachiaria brizantha* cv. Marandú. The treatments were as follows (i) *Erythrina berteroana*, (ii) *Inga edulis*, (iii) *Leucaena leucocephala*, and (iv) a no-tree, grass only control. Three replicates were established of each treatment, for a total of 12 experimental plots in a randomized blocked experimental design. In each replicate plot containing a tree treatment a total of 180 trees were arranged in three 0.5 by 7.5 m alleys and with the pasture grass. Stands of *I. edulis* and *L. leucocephala* had been established from locally collected seeds and *E. berteroana* planted from 1 m stakes 24-months prior to the experiment. At 18 months post planting all trees were pollarded to 2 m height.

Eight non-lactating *Bos taurus indicus* heifers that were visually estimated to be of equal size and body condition were selected for the browsing experiment. The cattle were initially placed in a training corral where they had a 15-day period of acclimatization. Electric fences prevented cattle from browsing adjacent plots. Permanent water (provided ad libitum) and 5 × 5 m shade stations were installed at the edge of each replicate and this area was excluded from all sampling.

**Figure 1:**
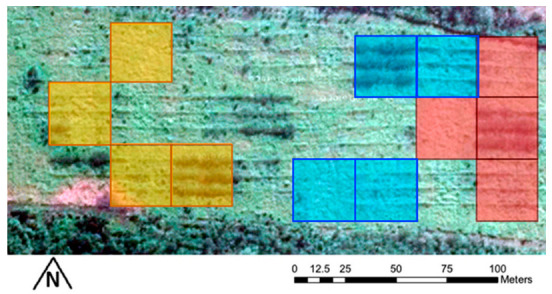
Satellite image and map of the random blocked experimental design for the cattle browsing trial. The trial was conducted from East to West in three phases, each of the four treatments (two cattle per plot) was replicated in each phase, phase 1 (red), phase 2 (blue) and phase 3 (yellow). (Map data: Google, Maxar Technologies).

The experiment began on the 13 February 2019. The eight cattle were randomly assigned to pairs for a total of four pairs of cattle. Each of the pairs were then moved into one of four treatment replicates (Phase 1) (Figure 1). After eight days, the same pairs of cattle were moved to the next randomly selected replicate where they remained for eight further days (Phase 2). Subsequently, the same pairs of cattle were moved into the last set of replicates where they remained for a further eight days (Phase 3). The experiment concluded on the 8 March 2019.

### 2.2. Forage Measurements

**Figure 2:**
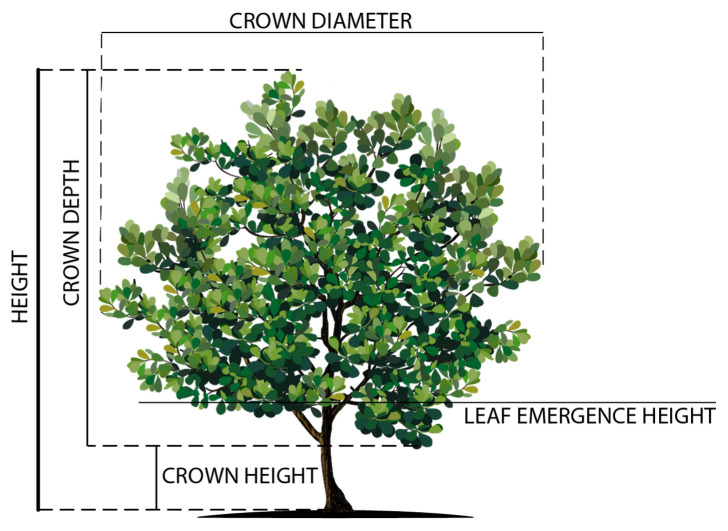
Diagram representing the location of measurements on tree, crown diameter, crown depth, crown height and height.

Within each replicate, thirty trees were selected from ten size classes. Tree height was measured from the ground to the top of the highest meristem (Figure 2). Leaf emergence height (LEH) was measured from the ground to where foliage volume assumed 5% of the total crown. A 5 m measuring tape was used to measure crown diameter (CD), taken as the mean of three horizontal measurements of the crown perpendicular to the ground. Crown radius (r) was calculated as the crown diameter divided by two. Crown depth (CD) was derived using Equation (1). Crown area (CA) approximates a circle and was derived using Equation (2). Crown volume (CV) was assumed to equate to a cylinder, the best fit for the pollarded trees, and was calculated using Equation (3):

CD = Height − LEH(1)

CA = πr^2^ (2)

CV = πr^2^CD (3)

The root collar diameter (RCD, measured at the widest part 0.05 m above the ground) of all trees within each plot was measured and divided into ten size classes. Six trees from each size class were randomly selected for destructive sampling of all leaves; three of which were harvested two days prior to the introduction of cattle, and three were harvested two days after the cattle had left. Tree foliage was harvested according to two height classes to allow for a comparison of the effect of resource height on resource intake. It was assumed that cattle could reach a maximum height of 1.6 m. For each tree, foliage higher than 1.6 m from ground level was harvested separately from foliage lower than 1.6 m. Foliage was oven dried at 70 °C until a constant weight was reached and reported as dry weight.

**Figure 3:**
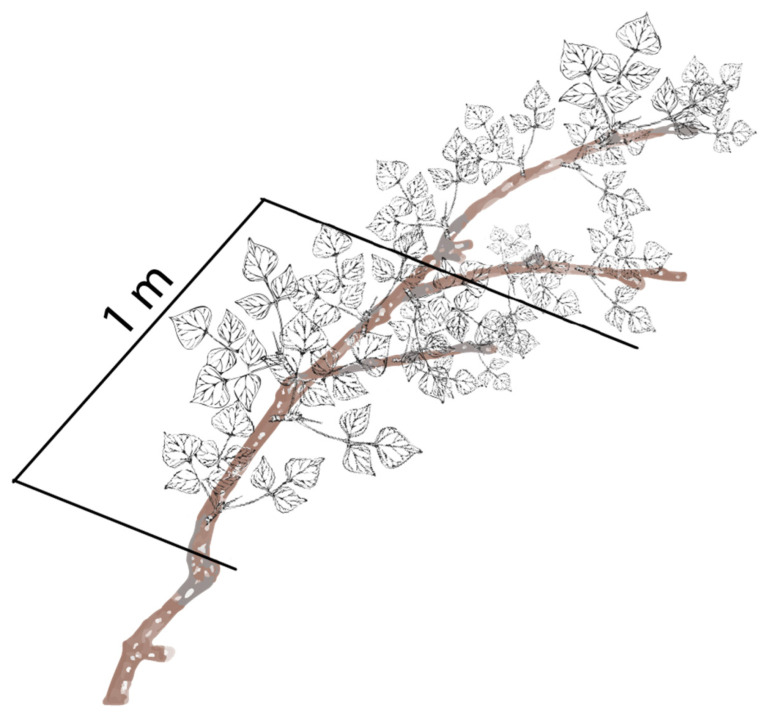
Diagram representing a section of branch with a 1 m section of leaves that were counted prior to and post the cattle trial.

Leaves were counted prior to and post the cattle trial to detect an effect of browsing on the number of leaves. Thirty trees from each replicate were chosen at random and two branches from each tree were chosen for leaf counts. The branches were marked in situ to highlight two sampling sections on each branch. The first section was measured from branches at a minimum height of 0.5 m from the ground. At 0.5 m a 1 m section was measured along the branch (Figure 3). This section is referred to hereafter as 0.5–1.5 m. The second section of branch was measured from a minimum height of 1.5 m for 0.5 m and is hereafter referred to as 1.5–2 m. All leaves within each section were counted. The replicates were subjected to direct cattle browsing within two days of sampling, and the sampling procedure was repeated on the same sections the day after the cattle left. The difference in the number of leaves prior to and post-trial was assumed to be due to utilization by cattle.

**Figure 4:**
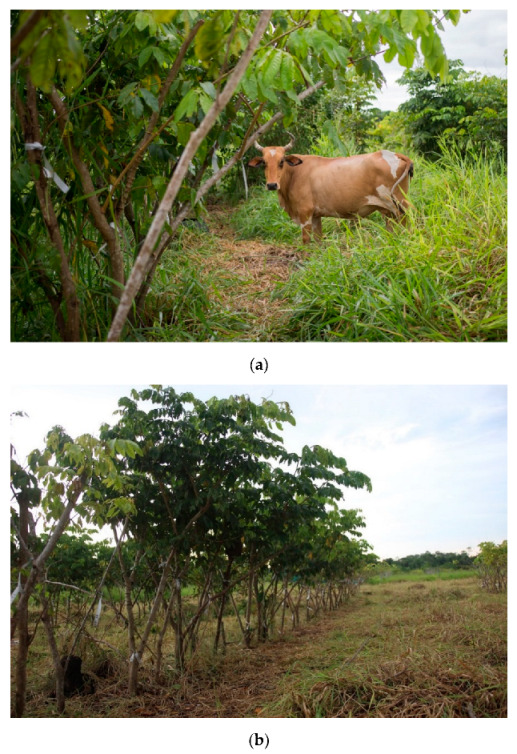
A photo taken from the same position of an *I. edulis* replicate on the first (**a**) and eighth (**b**) day of the cattle trial.

Immediately following each eight-day cattle trial, 30 trees in every replicate (20% of remaining trees) were randomly selected and visually assessed for signs of browsing or cattle impact (Figure 4). The height of the impact was recorded, and damage types were reported as either browsed or broken. Prior to and post browsing trial, all ground biomass was harvested within five randomly located 1 m^2^ quadrats. Sampling prior to the browsing trial subjectively considered biomass as either an ‘edible’ or ‘nonedible’ fraction. ‘Edible fraction’ was visually assessed as the leaf or stem (<25 mm) component of the grass no less than 10 cm from the ground. This fraction was considered the ‘leaf’ part of the grass most favoured by cattle. The inedible fraction was the remaining herbaceous biomass, which was of lesser nutritional value to the cattle as the aging grass had time to develop higher proportions of fibrous and structural compounds which are harder to digest [28]. Each fraction was weighed separately as fresh weight. Three subsamples were taken of each fraction and dried to a constant weight. The wet weight to dry weight ratio was then extrapolated to estimate the dry weight for the fresh biomass collected from the quadrats.

### 2.3. Statistics

All data were checked for normality (Shapiro–Wilk) and variance (Levene’s test). To test for a browsing effect on foliar biomass within species, the averages were compared prior to and post the cattle trial. The natural variation within the data resulted in a non-normal distribution that was not improved by log transformation and the data did not satisfy the requirements for parametric tests. The data did fulfil the requirements for non-parametric tests and therefore when the data (i) had comparable variances a Mann Whitney test was used (*I. edulis*, *L. leucocephala*), (ii) did not have comparable variances a Mood’s Median test was used (*E. berteroana*) [29].

The ratio of foliar biomass under and over 1.6 m was compared for each tree prior to and post the cattle trial to determine any effect of browsing height on intake of foliar biomass. The proportional change was then compared for each species using a proportional test. A one-way analysis of variance (ANOVA) was then used to test for differences between tree species. The LEH of *I. edulis* and *E. berteroana* prior to and post the cattle trial were compared within species using paired t-tests. *L. leucocephala* had a non-normal distribution but equal variance therefore a Mann–Whitney U test was employed. To detect effects of cattle browsing on crown volume; when the data displayed (i) homogeneous variance (*I. edulis* and *L. leucocephala*) a Mann–Whitney U test was used, (ii) nonhomogeneous variance (*E. berteroana*) a Kruskal–Wallis test was used.

The height of browsing impacts were compared for tree species using a one-way ANOVA and differences were determined using Tukey’s Honest Significant Differences test. Other browsing impact variables had non-normal distributions, so Kruskal–Wallis tests were used and differences in species were determined with Dunnett’s test. Dunnett’s test was used as it is considered an appropriate test for groups with unequal numbers of observations [30]. Mortality resulting from the browsing trial was calculated as the percentage change of individual trees prior to and post the trial. All statistical tests were carried out in R v3.2.2 [31].

## 3. Results

Cattle browsing reduced foliage biomass for all tree species. This effect was detected by all methodologies. The weight of dry foliar biomass harvested destructively below 1.6 m decreased for all species when compared prior to and post the browsing trial. *E. berteroana* showed a 99% decrease, *I. edulis* a 75% decrease and *L. leucocephala* an 80% decrease in biomass per tree (Table 1). A greater proportion of biomass was lost below 1.6 m than over 1.6 m for *E. berteroana* (*p* < 0.05), *I. edulis* (*p* < 0.01) and *L. leucocephala* (*p* < 0.01). When total foliar biomass (combined <1.6 m and >1.6 m) was considered, there was a reduction in total foliar biomass of *E. berteroana* of 92% (*p* < 0.01), 30% for *I. edulis* (*p* < 0.05) and an increase of 20% for *L. leucocephala* (*p* < 0.01). There were no differences detected on the removed foliar biomass at any or all heights between species.

Post-browsing, crown volume decreased by 67% for *E. berteroana* (*p* < 0.01), 51% for *I. edulis* (*p* < 0.01) and 31% for *L. leucocephala*, although the latter was not significant (*p* = 0.06) (Table 2). Leaf emergence height (LEH) increased for all species post-browsing, in *E. berteroana* by 49% (*p* < 0.01), in *I. edulis* by 50% (*p* < 0.001) and in *L. leucocephala* by 20% (*p* < 0.01).

The mean number of leaves decreased among all species as a result of cattle browsing (Table 3). Post-trial, *E. berteroana* had lost the greatest proportion of leaves in both the 0.5–1.5 m and the 1.5–2.0 m category (95% and 91%, respectively). *I. edulis* and *L. leucocephala* both lost more leaves under 1.5 m (80% and 87%, respectively) than over 1.5 m (48% and 80%, respectively).

**Figure A1:**
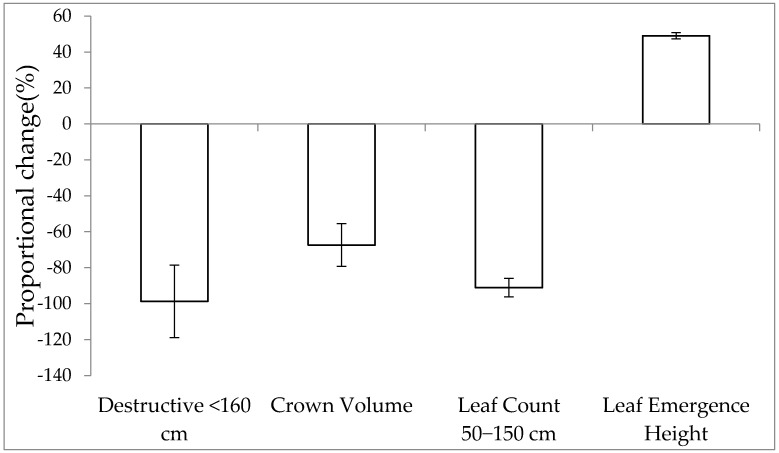
Proportional change (%) of *Erythrina berteroana* biomass for four assessment methodologies used to detect biomass removal following cattle browsing.

**Figure A2:**
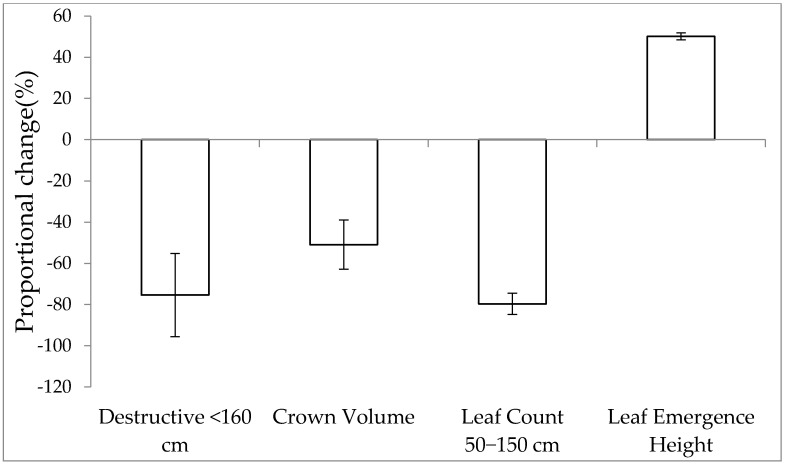
Proportional change (%) of *Inga edulis* biomass for four assessment methodologies used to detect biomass removal following cattle browsing.

**Figure A3:**
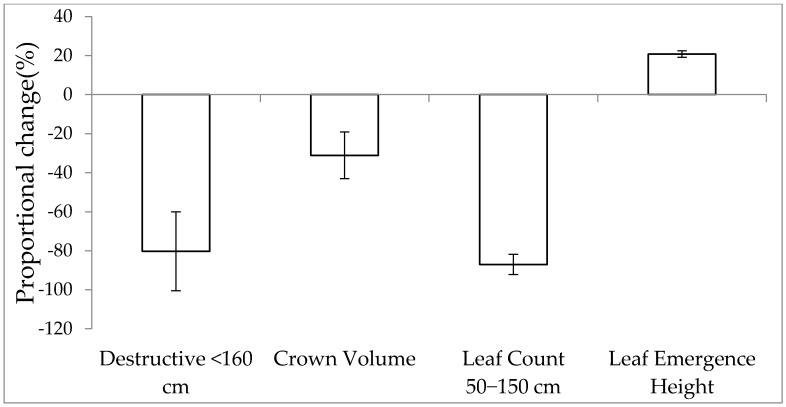
Proportional change (%) of *Leucaena leucocephala* biomass for four assessment methodologies used to detect biomass removal following cattle browsing.

When the methodologies were compared between species, the non-destructive estimate of leaf count at 50–150 cm showed comparable results to that of the destructive sampling <160 cm (Figure A1, Figure A2 and Figure A3).

Browsing damage was recorded on branches for all species (Table 4). The browsed branches of *E. berteroana* showed an increased diameter compared with other species. The branches of *E. berteroana* had a 35% greater diameter than *I. edulis* and 65% greater than *L. leucocephala*. There was no effect of species on maximum height of branches browsed. *E. berteroana* had the highest rate of mortality when measured immediately post browsing (12.5%), six times greater than that of the species with the lowest mortality, *I. edulis* (2.1%). *L. leucocephala* had a mortality of 4.8%.

**Figure 5:**
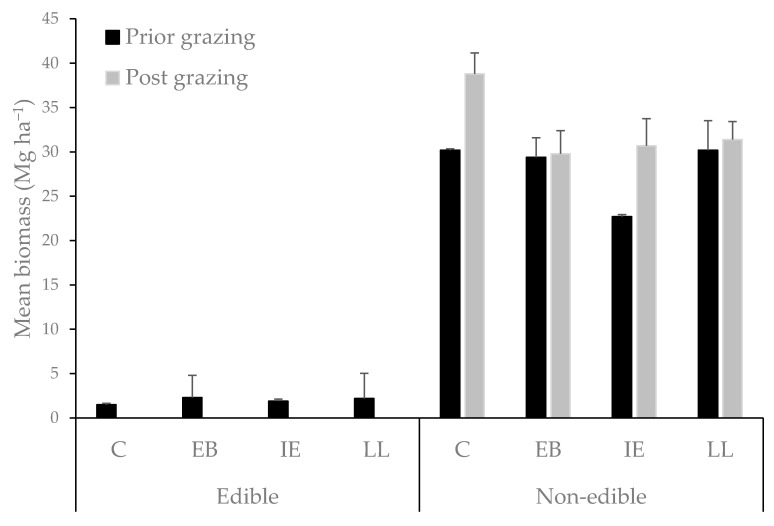
Mean average dry biomass of edible and non-edible fractions of grass (Mg ha^−1^) produced in the four treatments: *Erythrina berteroana* (EB), *Inga edulis* (IE), *Leucaena leucocephala* (LL), and a no-tree, grass only control (C), reported for destructive sampling carried out prior to and post the introduction of cattle. ‘Edible fraction’ was visually assessed as the leaf or stem (<25 mm) component of the grass no less than 10 cm from the ground. ‘Inedible fraction’ refers to the remaining fibrous and structural herbaceous biomass considered to be of lesser nutritional value to cattle. Error bars represent standard error values.

There were no differences between tree species on the amount of edible or non-edible herbaceous biomass available prior to the cattle trial (Figure 5). When herbaceous biomass was compared prior to and post the cattle trial, 100% of the edible fraction was consumed in all treatments. Combining the results of the edible fraction of grass with that of the destructive foliage < 1.6 m in each treatment as a conservative measure of total available edible foliage, the no-tree control treatment would produce 1.5 Mg ha^−1^, this was less than that produced by the tree treatments of *E. berteroana* (2.3 Mg ha^−1^), *I. edulis* (2.3 Mg ha^−1^) and *L. leucocephala* (2.2 Mg ha^−1^).

## 4. Discussion

The foliage of all tree species was palatable to cattle and no differences between species were detected in the amount of foliage consumed. Plots which included each of these three tree species produced more total available edible biomass than the grass monocultures. This study therefore demonstrates that silvopastoral systems in the Amazon using locally adapted tree legumes can increase per hectare productivity for cattle production when compared to conventional pasture-only grass monocultures. The increase in available edible biomass reported in this paper is a conservative estimate as this figure considered only the foliar biomass and only up to a height of 1.6 m. In practice, it was found that, by manipulating the branches, cattle were able to access browse up to a height of 2.6 m. When estimating the amount of tree foliage that is available for direct cattle browsing, resource height relative to the height of browsing is an important consideration [32]. Authors report maximum browsing heights of 1.9 m [33] to 2.2 m [34]. However, browse above this height was reached by cattle and therefore the results of this study underreport the total available forage.

*I. edulis* produced the most overall biomass and the most accessible foliage for the cattle of the tested species. Branch browsing and damage to branches as a result of cattle interaction was observed on *I. edulis*. However, *I. edulis* demonstrated the lowest mortality following cattle interaction of the tree species. This may be due to an increased diameter of *I. edulis* stems when compared with those of *E. berteroana* or *L. leucocephala.* Hohnwald et al. [35] reported that *I. edulis* was a ‘high’ producer of woody and leafy biomass, a frequently occurring tree in Capoeira, a nitrogen fixing legume and palatable to cattle. Hohnwald [20] reported that *I. edulis* had ‘bad performance’ on a scale of palatability and biomass, which suggested it had poor forage quality and was given an ‘intermediate’ value for mortality. However, the same study reported that *I. edulis* suffered from the overgrown grass sward during the establishment phase, but when established it was robust. The present study reports that *I. edulis* is a suitable candidate for further investigation for forage potential. To date, no studies have evaluated the effect of *I. edulis* consumption on cattle health. Dechnik-Vázquez et al. [16] ranked *I. edulis* 11th of 268 species by the number of times the species appeared browsed.

The highest nutrient concentrations have been found to occur in the most utilized and therefore compacted areas of Amazon pastures, for example, surrounding the water trough [36]. This leads to pasture degradation as nutrients are exported or leached from the soil [37]. It was observed that cattle spent time in the shade of *I. edulis* and studies have shown the roots of *I. edulis* can reverse soil compaction [8]. Therefore, it is possible that nutrient cycling may occur if nitrogen deposited in the shade of the trees becomes available to the animals via consumption of the foliage.

*E. berteroana* exhibited the greatest loss of foliar biomass following the trial, the largest diameter of branches consumed and broken, the greatest incidence of mortality, the highest mean and maximum point of branch browsing, and the greatest increase in leaf emergence height of the tree species. Unforeseen in the experimental design was that cattle would employ unique browsing strategies for each species. It was observed that the cattle displayed a browsing habit for *E. berteroana* of pulling down branches on which to browse. The branches would frequently break during this process and often sections of the broken branches would also be chewed and consumed. Post-trial, the majority of *E. berteroana* branches had been broken, in some cases entire plants were missing. The *E. berteroana* had been established through propagation by direct planting of 1 m stakes. This may have contributed to a substantially different growth form from that of *E. berteroana* when grown from seed. The 1 m stakes produced adventitious shoots formed from the callus tissue between the bark and the wood of the cut surface that may have resulted in weaker branches than an *E. berteroana* tree grown from seed. This may have increased the susceptibility of the trees to cattle damage.

Of the three tree species, *L. leucocephala* produced the least available foliar biomass. When pollarded to 2 m, *L. leucocephala* had produced adventitious buds at the point of pollarding, and therefore the majority of the foliar biomass was out of reach of the cattle. A pollarding height of 1 m may have been more appropriate to encourage leaf production at a height that would have made the foliage accessible to cattle. Guevarra et al. [38] found that optimal forage production was achieved through a planting arrangement of 15 × 50 cm and pollarding when the trees reached 1 m in height. The present study reports the ability of this species to grow in the Peruvian Amazon in association with *B. brizantha* cv. Marandú. *L. leucocephala* has been widely documented as a tropical forage tree but it has not yet been reported have potential for silvopasture in the Amazon. *L. leucocephala* is thought to be highly invasive [39]. In the 24 months following establishment, no incidences of natural regeneration of *L. leucocephala* were observed within the *B. brizantha* cv. Marandú pasture.

Destructive sampling was used to estimate the amount of foliage removed from the trees by direct cattle browsing. Samples were drawn from the same population and there was natural deviation within the samples of tree foliage that were harvested. An artefact of this natural deviation was a reported increase in total foliar biomass for *L. leucocephala*. This result highlights the challenges in conducting an experiment of this size and the need for continued development of methodologies to assess the availability of tree forage biomass to browsing cattle.

Silvopastoral systems present a ‘win-win’ opportunity for biodiversity conservation and poverty alleviation. Such systems can halt or reverse land degradation and, in the context of the Amazon, this may reduce the need for additional forest clearance. Despite the practical relevance of these systems, there is a paucity of empirical studies that evaluate the contribution of locally adapted trees and shrubs to per hectare productivity and the provision of ecosystem services. It is vitally important to support the transition to sustainable agriculture in the Amazon and future studies should work closely with local farmers and organisations to help develop strategies to adapt to and potentially mitigate the effects of climatic change.

## 5. Conclusions

The inclusion of locally adapted trees in pastures in the Amazon could provide an increase in per hectare productivity for livestock systems in the Amazon. *E. berteroana, I. edulis* and *L. leucocephala* were palatable to cattle when fresh and contributed to a higher total edible biomass than grass only monocultures. This study presents two local and novel tree legumes for consideration as forage trees in silvopasture, *I. edulis* and *E. berteorana*. Of the species in this study, *I. edulis* produced the greatest quantity of foliar biomass and demonstrated more resilience to cattle browsing than the other species and is recommended for further investigation as a forage tree. Further work is needed to understand the effects of these forages on animal health.

## Figures and Tables

**Table 1:** Mean dry foliar biomass per individual trees (g), standard deviation (SD) and number of individuals sampled (*n*) prior to and post cattle browsing either mean total tree foliar biomass or foliar biomass harvested < 1.6 m on potential browse species *E. berteroana*, *I. edulis* and *L. leucocephala*. Significant differences (*p* < 0.05) from prior to post browsing are denoted by an asterisk (*).

Species	Trial	*n*	Mean Total Foliar Biomass		Foliar Biomass <1.6 m	
			Mass (g)	SD	Mass (g)	SD
*E. berteroana*	Prior	87	45.3	66.2	15.5	66.2
	Post	90	3.8 *	11.8	0.2 *	11.8
*I. edulis*	Prior	88	297.1	347.7	141.7	347.7
	Post	90	204.5 *	348.2	34.9 *	348.2
*L. leucocephala*	Prior	87	60.4	193.0	6.6	193.0
	Post	90	72.3 *	277.9	1.3 *	277.9

**Table 2:** Mean and standard deviation (SD) of change per individual tree in crown volume (m^3^) and leaf emergence height (LEH, cm) prior to and post a cattle browsing experiment on individuals (*n*) of potential browse species, *E. berteroana*, *I. edulis* and *L. leucocephala*. Significant differences prior to and post browsing trial (*p* < 0.05) are denoted by an asterisk (*).

Parameter	Species	Prior to Trial			Post Trial		
		Mean	SD	*n*	Mean	SD	*n*
Crown Volume (m^3^)	*E. berteroana*	0.94	1.15	88	0.31 *	0.51	50
	*I. edulis*	2.01	2.05	48	1.02 *	1.65	46
	*L. leucocephala*	1.54	2.6	83	1.06	2.05	75
LEH (cm)	*E. berteroana*	112.6	40.1	88	167.8 *	74.2	50
	*I. edulis*	101.6	33.3	48	152.5 *	35.1	46
	*L. leucocephala*	191	45.9	83	230.7 *	53.5	75

**Table 3:** Mean leaf count (number of leaves per branch), standard deviation (SD) and mean reduction (%) prior to and post cattle browsing trial on individuals (*n*) of potential browse species *E. berteroana*, *I. edulis* and *L. leucocephala*. Significant differences (*p* < 0.05) between leaves counted prior to and post browsing are denoted by an asterisk (*).

Sampling Range	Species	Prior/Post	*n*	Number of Leaves Per Branch	SD	Mean Reduction (%)
1.5–2 m	*E. berteroana*	Prior	65	26.3	18.6	
		Post	65	1.4 *	3.5	−95
	*I. edulis*	Prior	70	17.6	18.8	
		Post	70	9.2 *	14.4	−48
	*L. leucocephala*	Prior	37	16.1	14.4	
		Post	37	3.9 *	7.2	−76
0.5–1.5 m	*E. berteroana*	Prior	132	29.3	23.6	
		Post	132	2.6 *	4.6	−91
	*I. edulis*	Prior	163	24.6	23.2	
		Post	163	5.0 *	6.6	−80
	*L. leucocephala*	Prior	99	16.2	13.6	
		Post	99	2.1 *	5.0	−87

**Table 4:** Impacts measured following a cattle browsing trial on 30 individuals of each of three tree species *E. berteroana, I. edulis* and *L. leucocephala*. Impact types were classified as browsing-type damage (browsed) or breakage-type damage (broken) of two categories, branches (all species) and leaves (*I. edulis*). Mean diameter (mm), mean height (cm) and maximum height (cm), as well as respective standard deviations (SD) reported on a number (*n*) of individual trees. Significant differences between species (*p* < 0.05) are denoted by an asterisk (*).

Species	Impact Category	Impact Type	Diameter (mm)		Height (cm)			
			Mean	SD	Mean	Max	SD	*n*
*E. berteroana*	Branch	Broken	12.8 *	7.2	153	240	41.7	126
	Branch	Browsed	5.0 *	1.8	156	256	42.7	222
*I. edulis*	Branch	Broken	9.0 *	4.7	134	195	37.0	39
	Branch	Browsed	3.9 *	1.4	150	246	36.3	181
	Leaf	Browsed	2.5	0.7	144	197	55.3	245
*L. leucocephala*	Branch	Broken	6.5 *	3.8	135	260	44.1	41
	Branch	Browsed	2.8 *	0.9	154	225	36.2	113

## Data Availability

No new data were created or analyzed in this study. Data sharing is not applicable to this article.
